# Investments

**Published:** 1873-05

**Authors:** 


### INVESTMENTS.

There are two sisters living in a handsome brown stone house in one of the cross streets up-town, near Fifth avenue. They are boarders; they had their own private table and drove in their own carriage, with every other comfort that a large income could procure. Yesterday the carriage and the splendid horses were sold at auction; to-morrow they leave the luxurious home of sixteen years, and at the ages of sixty-six and sixty-nine, they go out into the great world, paupers. It happened thus: Their father had been an honorable and successful New York merchant, and dying a widower, with two only children, girls, left for them a large fortune, in the hands of an executor, a lawyer, of spotless reputation and of a legal ability so acknowledged, that he gradually fell into an exclusive line of business, adjudicating differences between gentlemen who respected themselves and their purses too much to go to law. Not long ago this gentleman died. We have been to his house; he gave us the idea of a kindly old man. The press of the city was lavish in his praise; for in seventy years of life in New York, there never was the shadow of a suspicion against his fair name, and even now it is not known that he ever did a wrong thing, except this one—he paid these ladies the interest on their fortune to the day and hour always, but no more than the interest, so as to leave the entire principal for his own use; on the day he died, there was not a dollar left of the one hundred and ninety-two thousand which his old friend had left in his care for his two orphaned daughters.

Yesterday this thing came to our knowledge. A young lady had been traveling abroad for several years with a gentleman and two other ladies of my family connection, so the narration is personally known to be according to the facts stated. On returning to New York she married, and her young husband, being a business man, thought proper to investigate the affairs of her father's estate, and calling upon the executor, ascertained that he had spent, for his own purposes, fifty thousand dollars of his young wife's money. It gradually became noised abroad that he had been made trustee and agent and executor in various other cases; in every instance the money was gone. So great was the confidence reposed in him, that quite a number of persons among the old Dutch families had selected him as their executor. It is not known that he was a member of the church; very likely he was, and a teacher, if not superintendent in the Sunday school; may be an elder or deacon or trustee or vestryman; certain it is he was "Scoundrel in Chief," the "Head Center" of thieves. Whatever his offices were he resigned them all promptly. That admirable people, "The Society of Friends," have a most excellent mode of procedure when one of their number fails in business; his books are promptly called for; immediate and searching investigations by accountant experts are instituted; if everything is on the square, they furnish him with money enough to go ahead again; for these Quakers have always plenty of money, and their motto is: "Help the helpless and the unfortunate," and they always make money by it, for if a member gets poor and thriftless, they make it a matter of principle to take kindly care of him until he dies; but this requires money, which they have to earn. Suppose, however, by a little help and encouragement and moral countenance, they put a business man on his feet again, he not only repays them, not only saves them from supporting him, but is able to help to take care of others. The "world's people" uniformly give a broken merchant a parting kick, never caring to take the trouble to ascertain whether his failure was a misfortune or a fraud. But woe be to the fellow whose books show even negligence, let alone downright, deliberate wrong doing; he is hustled over the fence of the fold, neck and heels, and is no longer one of them, nothing but an heathen man and a "sinner."

A case in point occurred many years ago in Philadelphia. There was a man, named Mickel Whitall; don't know whether he is alive or not now; hope he is, for he, and such as he, are greatly needed in these degenerate days. He had been in business a number of years; industrious, just, frugal and moderate in all his desires; but he failed: everybody was surprised, sorry; for he was known everywhere as "a good man and a just." His books were called for; there was no halting and hanging back, as is the fashion nowadays; but they were promptly produced; vouchers were forthcoming for everything; there were no erasures, no tinkerings, no leaves missing, everything was perfectly open, straight and fair. But it did not end here. Men had failed to pay what they justly owed for goods furnished; the point to be determined was this: Did Mickel Whitall credit doubtful men? did he recklessly trust for goods, for which others had trusted him? for if it were found that he had not used reasonable caution in this direction, he must go overboard. All his defaulting creditors were found to be men of good reputation, and had been, as the confession of Faith of Friends requires, living within the bounds of their income—for this question is put "in meeting" every year to every adult Friend. So the committee reported that Mickel Whitall was an honest man and should be appointed a committee of one to wind up the business of the concern and go ahead again. So Mickel went about the work with a will; found that he had been more scared than hurt, paid all his debts, principal, interest and *damages!* and had fifty thousand left; made money faster than before; raised a family of daughters, who got good husbands, made themselves notable in good doing, and to this day are adding honors to the Whitall stock.

But let us go back to the subject of investments, first begging pardon of our "Friend," if he be still in the land of the living, and make a suggestion which may be of practical value. Parents in this country will continue to work themselves into premature graves to make and leave fortunes to their children, oftentimes to their own temporal and eternal undoing; the point is, to inquire how to invest money safely, and to be sure, as sure can be, in this changing world, that children will have the benefit of it as long as they live. It is perfectly dreadful to think of a man working hard all his life for his wife and children and then for some one else to spend the money, while they are left to struggle on in poverty to the latest day of life; there could be no more impressive illustration of the scripture truth, "All is vanity and vexation of spirit." The safest, surest and least troublesome investment is what is known as government securities, "registered," which means, that the United States will borrow any amount of money from you, give you a note for it, and pay you the interest quarterly, as long as you want to live. But this note might be lost, or mislaid or burned up; what then? There is a "register" kept in Washington City of the names and amounts of all such notes and who own them; inform them of the loss and they will give you a new note for it; and nobody can draw the interest but the real owner as long as the note is held in his name. Another plan is, leave all your money to some church organization, the interest to be paid to your children in person or to their order, as long as they live, and then the principal to go to that church to be distributed to its various beneficent institutions; then the money will be in the care of several persons, and as all of the principal will revert to the church, the certainty of its being taken good care of is largely increased; and then of your children it will be said: "Their bread shall be given them, and their water shall be sure." As to the grandchildren, let them take care of themselves. If their parents are not thrifty enough to save up something for them from the income you have left them and from their own personal exertions and economies, the breed is not of much account, and the sooner it becomes extinct the better for the country and the church.

The truth is, your grandchildren will be much more certainly saved from want and destitution and misery by your making the church your residuary legatee, than by giving everything you have out and out to your children. The churches, in large cities more especially, are fast arranging to have "Homes" for their old and poor and unfortunate. There is a "Presbyterian Home" in Philadelphia, from which however no one is turned away; it is for every color, cast and creed.

There is another such in New York to which that steward of the Lord,

THE LENOX, has given half a million of dollars or more. Every inmate is allowed to pay much or nothing, according to pecuniary ability, so that no respectable Presbyterian in the City of New York need ever fail to find a room and bed and table. The ladies of Dr. Chapin's church, in their universal large-heartedness, are laboring hard to establish a home for members of the Universalist church, and it is now already in operation.

Or if any one is afraid that the church will get too much money in its efforts to save the whole human family from the degradation of lust and sin and heathenism, to the liberty and elevation and light of the gospel, the same fund can be left to some chartered body as a hospital, or college, or seminary, for any such body of men is safer than an individual.

The lesson is: Invest safely for your children, or they will come to poverty and disease and broken hearts and premature graves. An abiding feeling of being comfortably provided for, for life, without a peradventure, is one of the best promoters of longevity ever yet known.

